# Long short-term memory (LSTM) recurrent neural network for muscle activity detection

**DOI:** 10.1186/s12984-021-00945-w

**Published:** 2021-10-21

**Authors:** Marco Ghislieri, Giacinto Luigi Cerone, Marco Knaflitz, Valentina Agostini

**Affiliations:** 1 Department of Electronics and Telecommunications, Politecnico Di Torino, 10129 Turin, Italy; 2 PoliToBIOMed Lab, Politecnico di Torino, 10129 Turin, Italy; 3 Laboratory for Engineering of the Neuromuscular System (LISiN), Departement of Electronics and Telecommunications, Politecnico di Torino, 10129 Turin, Italy

**Keywords:** Deep learning, EMG, EMG-based interfaces, Gait analysis, Muscle activity, Muscle activation intervals, Onset-offset detection, Surface electromyography, RNN

## Abstract

**Background:**

The accurate temporal analysis of muscle activation is of great interest in many research areas, spanning from neurorobotic systems to the assessment of altered locomotion patterns in orthopedic and neurological patients and the monitoring of their motor rehabilitation. The performance of the existing muscle activity detectors is strongly affected by both the SNR of the surface electromyography (sEMG) signals and the set of features used to detect the activation intervals. This work aims at introducing and validating a powerful approach to detect muscle activation intervals from sEMG signals, based on long short-term memory (LSTM) recurrent neural networks.

**Methods:**

First, the applicability of the proposed LSTM-based muscle activity detector (LSTM-MAD) is studied through simulated sEMG signals, comparing the LSTM-MAD performance against other two widely used approaches, i.e., the standard approach based on Teager–Kaiser Energy Operator (TKEO) and the traditional approach, used in clinical gait analysis, based on a double-threshold statistical detector (Stat). Second, the effect of the Signal-to-Noise Ratio (SNR) on the performance of the LSTM-MAD is assessed considering simulated signals with nine different SNR values. Finally, the newly introduced approach is validated on real sEMG signals, acquired during both physiological and pathological gait. Electromyography recordings from a total of 20 subjects (8 healthy individuals, 6 orthopedic patients, and 6 neurological patients) were included in the analysis.

**Results:**

The proposed algorithm overcomes the main limitations of the other tested approaches and it works directly on sEMG signals, without the need for background-noise and SNR estimation (as in Stat). Results demonstrate that LSTM-MAD outperforms the other approaches, revealing higher values of F1-score (F1-score > 0.91) and Jaccard similarity index (Jaccard > 0.85), and lower values of onset/offset bias (average absolute bias < 6 ms), both on simulated and real sEMG signals. Moreover, the advantages of using the LSTM-MAD algorithm are particularly evident for signals featuring a low to medium SNR.

**Conclusions:**

The presented approach LSTM-MAD revealed excellent performances against TKEO and Stat. The validation carried out both on simulated and real signals, considering normal as well as pathological motor function during locomotion, demonstrated that it can be considered a powerful tool in the accurate and effective recognition/distinction of muscle activity from background noise in sEMG signals.

**Supplementary Information:**

The online version contains supplementary material available at 10.1186/s12984-021-00945-w.

## Background

Dynamic muscle activity can be non-invasively investigated through surface electromyography (sEMG). Determining the start (onset) and end (offset) time-instants of muscle activations during human movements is of great interest in different research fields, such as gait analysis [1], motor rehabilitation and sport science [2], myoelectric control of prostheses [3], human–machine interaction [4], design of biofeedback systems [5], and pre-processing of muscle synergy extraction [6–9]. In particular, the accurate temporal analysis of muscle activation in terms of burst onset, duration of the activation interval, and burst offset, can be useful in the assessment of the altered locomotion patterns of orthopedic and neurological patients [10, 11].

In an attempt to increase the accuracy of the temporal analysis of muscle activations, several methods have been proposed in the literature, from the simplest approaches based on single-threshold detectors [12] to more complex approaches based on wavelet transform [13, 14], statistical optimal decision criteria [15, 16], or deep learning techniques [17–26]. One of the most widely used ways to detect the timing of muscle activations from sEMG signals is using a double-threshold detector, such as the double-threshold statistical detector by Bonato et al*.* [27], specifically developed for gait analysis. The authors of that paper claims an estimation bias on the onset timing of less than 10 ms, evaluated on simulated signals. However, this detector requires, as a necessary input parameter, to set the first (amplitude) threshold, the background-noise power. Furthermore, to fine-tune the second (temporal) threshold, it is important to estimate the Signal-to-Noise Ratio (SNR), i.e., through the algorithm described in [28]. Another standard approach is the single-threshold detector based on the Teager–Kaiser Energy Operator (TKEO) [29, 30], where the reported onset bias is 13 ms and 55 ms on simulated and real sEMG signals, respectively.

The majority of the existing threshold-based methods suffers from two main limitations. First, the selection of the muscle activation intervals is usually based on the extraction of some time- or frequency-domain features (i.e., the signal amplitude or energy, SNR, …) which may not be sufficient to properly detect the onset/offset time instants. Threshold-based algorithms usually rely only on the signal energy or amplitude to detect muscle activation intervals, while ignoring many other features that might lead to a more accurate temporal analysis of the dynamic muscle activity. Second, since the amount of noise superimposed to the sEMG signals may vary during the recording sessions due to changes in the skin–electrode interface characteristics or in the ground reference level, the performance of the threshold-based approaches may be strongly affected. To the best of the authors’ knowledge, only a few studies have been focused on the definition of models able to efficiently work even at very low SNR values [13] or designed to deal with changes in the SNR of the sEMG signal over time [31]. The detection of the start and end time-instants of muscle activations during human movements can also be heavily compromised by the presence of spurious background spikes, both in physiological and pathological conditions, which may have different sources, such as the hyper-excitable motor unit discharges characterizing stroke survivors or spinal cord injury patients and a slight displacement of the skin–electrode interface [32–34].

Alternative methods, such as deep learning approaches, are being explored to perform sEMG-based pattern recognition [17–25]. The topic dealt with in this paper is somewhat easier compared to a pattern-recognition problem. Indeed, we are not interested in classifying different movements, but simply detecting the presence or absence of muscle activation. Exploiting artificial intelligence, such as a recurrent neural network (RNN), resulted a winning strategy in a wide variety of different applications and might be explored also for our problem. RNN is a powerful learning algorithm inspired by the biological neural networks that constitutes the human brain, and it is trained to present to the network a large number of labeled “examples” [35]. More specifically, long short-term memory (LSTM) neural networks are a widely used type of RNNs designed to recognize patterns and time-dependencies in sequential data, such as numerical time series, texts, and audio tracks [36]. These neural networks were first introduced by Hochreiter and Schmidhuber in 1997 [37] and represent an extension of the recurrent neural networks (RNNs), allowing for a better assessment of the time-dependencies in long sequential data. Nowadays, even if LSTM recurrent neural networks represent the state of art in natural language processing and speech recognition problems [38], no studies applying these recurrent neural networks to the muscle activity detection problem have been published in the literature.

Due to the time-series nature of the sEMG signals, LSTM recurrent neural networks could be applied for identifying the muscle activity time-instants without relying on the selection and extraction of heuristic features from sEMG signals.

This contribution aims at assessing the applicability of a novel approach for muscle activity detection, based on LSTM recurrent neural networks, specifically developed to overcome the main limitations of the standard approaches. The performance of the LSTM-based Muscle Activity Detector (LSTM-MAD) is evaluated and compared against two of the most widely used approaches: a standard approach (single-threshold detector TKEO) [29, 30] and a statistical approach (double-threshold statistical detector Stat) [27] in terms of precision, recall, F1-score, Jaccard similarity index, and onset/offset bias both on simulated and real sEMG signals.

## Methods

**Fig. 1:**
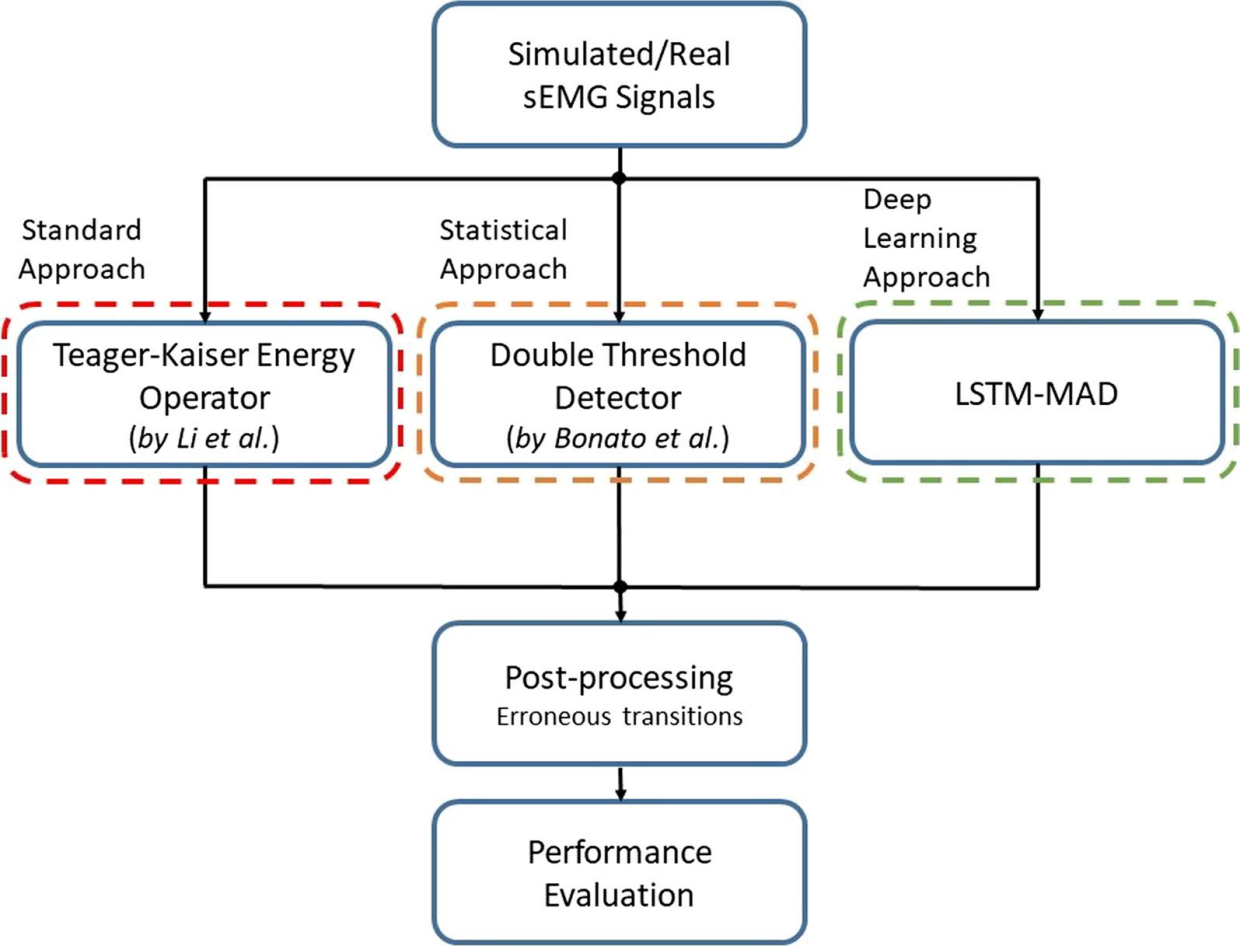
Block diagram of the procedure followed to assess the performance of the new LSTM-MAD (LSTM-based Muscle Activity Detector) compared to standard and statistical approaches for muscle activity detection

First, a dataset of simulated sEMG signals was built to assess the applicability of the LSTM-based approach to muscle activity detection and to compare its performance against the two muscle activity detectors TKEO [29, 30] and Stat [27]. Second, we further compared the performance of the three detectors on simulated sEMG signals, specifically highlighting the effect of the SNR of sEMG signals. Finally, LSTM-MAD was applied also to real sEMG signals, acquired from lower limb muscles during gait, to emphasize its advantages also in real contexts. Figure 1 represents the block diagram of the procedure followed in this study. Each block is described in the following paragraphs.

### Simulated data

The sEMG signals acquired during cyclic movements, such as walking, cycling, and running, can be modeled by the superimposition of two different contributions: (i) the electrical activity (*s*) generated by each muscle during the contraction and (ii) the background noise ($n$) mainly generated by the neighboring muscles, the features of the electrode–skin interface, and the acquisition system electronics. Under the hypothesis of cyclic contractions, the sEMG signal can be defined as a cyclostationary process [39] and, therefore, described through the superimposition of two different stationary processes [28]:

i. The muscle activity ($s$) modeled as a Gaussian process with zero-mean and variance ${\sigma }_{s}^{2}$, as described in (1):
  1 $$s\left(t\right)\in N\left(0,{\sigma }_{s}^{2}\right)$$
  where ${\sigma }_{s}$ was set equal to ${10}^{({SNR}/20)}\cdot 1\,\upmu V$;
ii. The background noise ($n$) modeled as a zero-mean Gaussian process with variance ${\sigma }_{n}^{2},$ as described in (2):
  2 $$n\left(t\right)\in N(0,{\sigma }_{n}^{2})$$
  where ${\sigma }_{n}$ was set equal to $1\mu V$.

Each realization of the muscle activity process $s\left(t\right)$ was simulated assuming a time period of 1 s (i.e., the gait cycle duration) and a sampling frequency of 1 kHz [28]. Physiological muscle activity was modeled by time-windowing the Gaussian process $s(t)$ through a single truncated Gaussian function centered at 50% of the gait cycle [28]. Different standard deviations (*σ*) and time supports ($2\alpha \sigma$) of the truncated Gaussian function have been considered to simulate sEMG signals similar to those observed in leg, thigh, and trunk muscles during gait. More specifically, three different values of the standard deviation ($\sigma$ = 50, 100, and 150 ms) and four different values of the time support $2\alpha \sigma$ (with $\alpha$ = 1, 1.5, 2, and 2.4) have been tested [27]. Then, the background noise process ($n\left(t\right)$) was added. Nine different values of Signal-to-Noise Ratio (SNR) were simulated (SNR = 3, 6, 10, 13, 16, 20, 23, 26, and 30 dB) [27]. For each triplet of $\sigma$, $\alpha$, and SNR values, 100 different realizations have been simulated and, therefore, a dataset composed by 10,800 different realizations (3 standard deviations × 4 time supports × 9 SNRs × 100 realizations) was built.

**Fig. 2:**
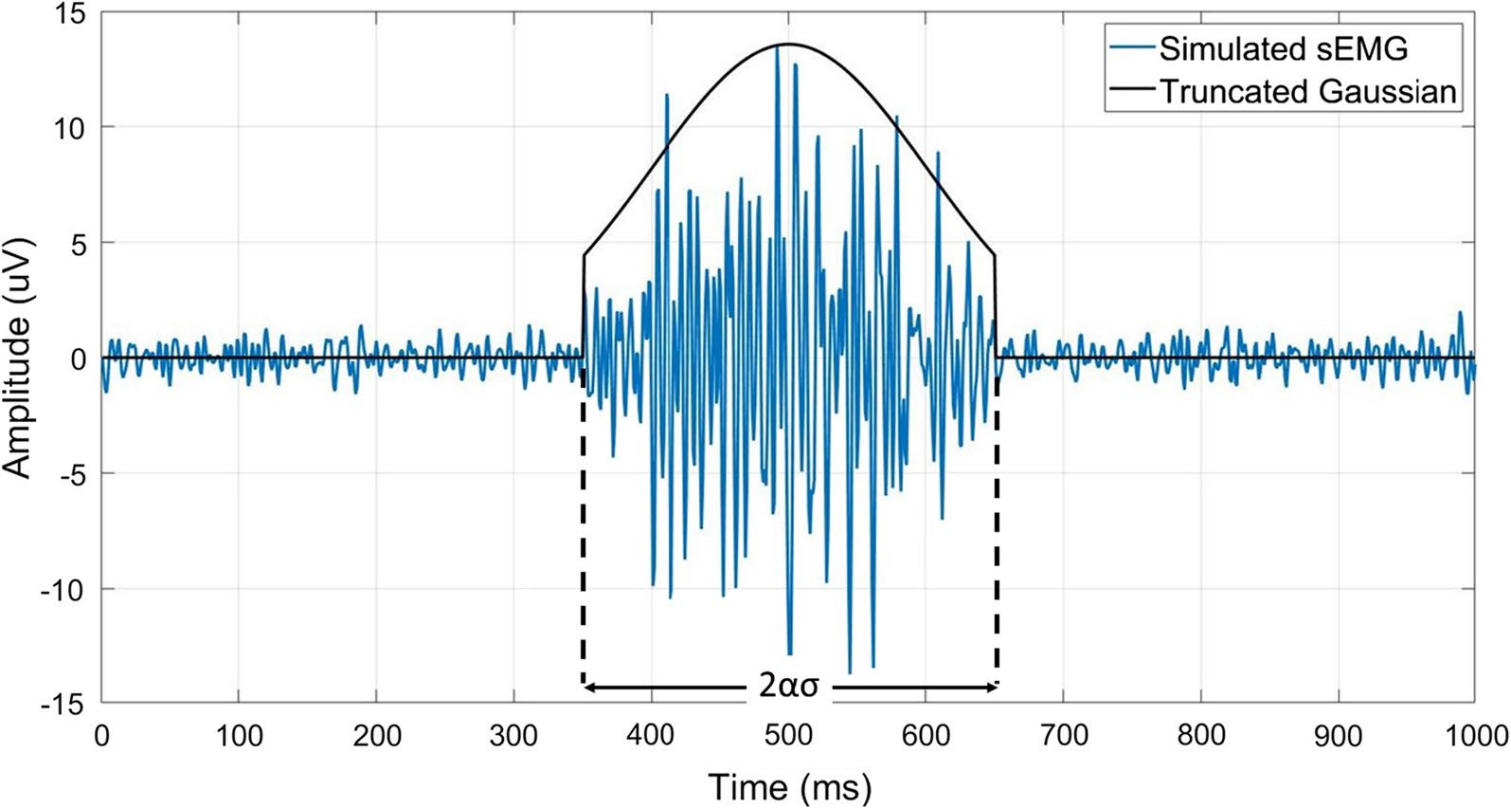
Example of a simulated sEMG signal (blue line) with the indication of the truncated Gaussian function (black line) used for the simulation of the muscle activity. The SNR is set equal to 20 dB, the standard deviation of the truncated Gaussian (σ) is equal to 100 ms, and the time support (2ασ) is obtained for α = 1.5

The simulated sEMG signals were then band-pass filtered through a 4^th^ order Butterworth digital filter with a lower cut-off frequency of 10 Hz and a higher cut-off frequency of 450 Hz [40]. Figure 2 represents an example of a simulated sEMG signal with the superimposition of the truncated Gaussian function $s(t)$ used to model the physiological muscle activity.

The time-instants relative to each simulated muscle activity ($s$) were defined by a binary mask (${y}_{Sim}$) that was set equal to 1 in correspondence of the time-instants in which the truncated Gaussian assumed values higher than 0, and it was set equal to 0 otherwise.

### Real data

Gait data acquired from 20 subjects were retrospectively analyzed to test the performance of the three different approaches when applied to real sEMG signals. Subjects were randomly selected from our database to include both healthy individuals and patients affected by neurological or orthopedic pathologies, during walking tasks [8, 41, 42]. This non-homogeneous group of subjects was specifically chosen to verify that the algorithm works under different conditions. In particular, eight out of 20 subjects were healthy adults (healthy age: 38.0 ± 13.1 years, height: 164.9 ± 5.4 cm, weight: 65.4 ± 21.2 kg) [8], six were patients after unilateral Total Hip Arthroplasty (THA age: 73.8 ± 8.4 years, height: 175.5 ± 7.6 cm, weight: 86.8 ± 16.3 kg) [41], and the other 6 were patients affected by idiopathic normal pressure hydrocephalus (NPH age: 75.7 ± 6.3 years, height: 170.5 ± 6.3 cm, weight: 72.5 ± 10.4 kg) [42].

Gait data were recorded through a multichannel acquisition system (STEP32, Medical Technology, Italy) [8, 43, 44]. SEMG signals were acquired through active probes (configuration: single differential, size: 19 mm × 17 mm × 7 mm, Ag-disks diameter: 4 mm, interelectrode distance: 12 mm, gain: variable in the range from 60 to 86 dB) placed over the following 4 muscles of the lower limb: rectus femoris (RF), lateral hamstring (LH), lateral gastrocnemius (LGS), and tibialis anterior (TA). Active probes were positioned according to the guidelines suggested by Winter [45]. Details on the sEMG sensor placement are described in a previous work by Agostini et al. [44]. The dominant lower limb (i.e., the leg used to kick the ball or to start walking) was analyzed for healthy subjects, while the clinically most affected limb was selected for pathological patients. Before applying the tested detectors, the acquired sEMG signals were band-pass filtered through a 4^th^ order Butterworth digital filter with a lower cut-off frequency of 10 Hz and a higher cut-off frequency of 450 Hz [40].

For each subject, 5 gait cycles were randomly selected from the whole walking task to build the real dataset. Therefore, a dataset composed of 400 different sEMG signals (20 subjects × 5 gait cycles × 4 muscles) was obtained. The time-instants relative to each real-muscle activation were visually segmented by three experienced operators through a custom MATLAB® GUI (graphical user interface). More specifically, a binary mask (${y}_{Real}$) was set equal to 1 in correspondence of the time-instants in which the majority of the expert operators (at least two out of three) detected a muscle activity and to 0 otherwise.

### Standard approach: Teager–Kaiser Energy Operator (TKEO)

One of the most commonly applied standard approaches for muscle activity detection is the Teager–Kaiser Energy Operator (TKEO), which has been demonstrated to increase the accuracy of the simple linear envelope approach [29, 30].

More specifically, a single-threshold was applied to the sEMG signals after the computation of the TKEO (ψ), defined as in (3):

3 $${\psi }_{x(n)}= {x(n)}^{2}-x\left(n+1\right)x(n-1)$$

where $x$ represents the sEMG time-series and $n$ the sample number. The single threshold was defined as described in (4):

4 $$Th= {\mu }_{n}\pm j\times {\sigma }_{n}$$

where ${\mu }_{n}$, ${\sigma }_{n}$ and $j$ represent the mean of the background noise, the standard deviation of the background noise, and a multiplicative constant, respectively. In this study, the constant $j$ was set equal to 7 as suggested in [29, 46]. Since the average (${\mu }_{n}$) and the standard deviation (${\sigma }_{n}$) of the background noise are required as inputs of this approach, the time-instants corresponding to the noise were automatically selected considering those that were simulated or segmented as background noise for the simulated and real sEMG signals, respectively.

The output of this detector was finally defined as a binary mask (${\widehat{y}}_{TKEO}$) that was defined as follows:

- $${\widehat{y}}_{TKEO}$$ = 1, if $${\psi }_{x(n)}$$ ≥ $$Th$$;
- $${\widehat{y}}_{TKEO}$$ = 0, if $${\psi }_{x(n)}$$ <$$Th$$.

### Statistical approach: double threshold statistical detector (Stat)

The statistical approach used in this study is the double-threshold statistical detector proposed in [27]. The principal computation steps are the following:

i. An auxiliary sequence ${z}_{i}$ is computed from the sEMG signals as the sum of the squared values of two successive samples (5)
  5 $${z}_{i}= {x}_{i}^{2}+{x}_{i+1}^{2}$$
  where ${x}_{i}$ and ${x}_{i+1}$ represent two consecutive samples of the sEMG time series;
ii. A first (amplitude) threshold ζ is applied on a sliding detection window defined by *m* consecutive samples of the auxiliary sequence ${z}_{i}$;
iii. Muscle activation is detected if at least ${r}_{0}$ (temporal threshold) out of *m* consecutive samples of the sliding detection window are equal to or above the first threshold ζ.

In this study, the length of the observation window (*m*) was set equal to 5, while the temporal threshold ${r}_{0}$ was set equal to 1 [27].

The output of this detector was finally defined as a binary mask (${\widehat{y}}_{Stat}$) that was defined as it follows:

- ${\widehat{y}}_{Stat}$ = 1, if ${z}_{i}$ ≥ ζ for at least ${r}_{0}$ out of *m* samples;
- $${\widehat{y}}_{Stat}$$ = 0, otherwise.

### Deep learning approach: LSTM-MAD

An LSTM recurrent neural network model is generally composed of the following architecture:

i. An input sequence layer;
ii. One or more LSTM layers used to learn the time-dependencies within the sequential data;
iii. A fully connected layer used to convert the output size of the previous layers into the number of classes to be recognized;
iv. A softmax layer used to compute the belonging probability to each class;
v. A classification output layer used to compute the cost function.

In this study, several LSTM recurrent neural network models were tested to assess the applicability of the proposed approach for muscle activity detection, considering direcly sEMG signals, without any feature extraction step. To define the best LSTM model for muscle activity detection, each of the two datasets of simulated and real sEMG signals was divided into 3 different sets: training set (70%), validation set (15%), and test set (15%), respectively. The training set was used to train the LSTM models, while the validation set (or development set) was used to evaluate the network performance and to avoid the overfitting of the training data. More specifically, the validation set was used to stop training automatically when the validation accuracy stopped increasing to avoid overfitting [35]. Finally, the test set was used for the final validation and the comparison of LSTM-MAD with the other two detectors.

Using the Deep Learning Toolbox of MATLAB® release R2020b (The MathWorks Inc., Natick, MA, USA), 720 different LSTM models were tested. All the LSTM models had a sequence input layer consisting of 1 unit (i.e., the dimension of a single simulated or real sEMG signal) and a fully connected output layer consisting of 2 units (i.e., the number of classes to be recognized). Different numbers of LSTM hidden layers (*n*), numbers of hidden units for each LSTM hidden layer (${n}_{units}$), learning rate ($\alpha$) values, and drop period ($\delta$) values were tested to achieve the LSTM architecture with the highest performance. More specifically, two different number of LSTM hidden layers ($n$ = 1 and 2), nine different numbers of hidden units for each LSTM hidden layer (${n}_{units}$= 100, 125, 150, 175, 200, 225, 250, 275, and 300), five different learning rates ($\alpha$= 0.01, 0.015, 0.02, 0.025, and 0.03), and eight different drop rate values ($\delta$ = 10, 15, 20, 25, 30, 35, 40, and 45) were tested [35]. The adaptive moment (ADAM) optimization algorithm was adopted in this work to train all the tested LSTM models [47]. The performance of each LSTM model was assessed considering the simulated (or real) test set by computing the overall classification accuracy, defined as the number of correctly classified sEMG samples normalized to the total number of sEMG samples within the test set.

The training process was performed on a workstation with a 3.2 GHz six-core CPU, 32 GB of RAM memory, and a 64-bit Windows operating system.

The ${y}_{Sim}$, extracted from the truncated Gaussian functions, and the ${y}_{Real}$, manually defined by the expert operators through the MATLAB® GUI, were used as target (or ground truth) to train and test each LSTM model for the simulated and real datasets, respectively.

The output of the LSTM approach was computed as a binary mask (${\widehat{y}}_{LSTM-MAD}$) that was defined as it follows:

- ${\widehat{y}}_{LSTM-MAD}$ = 1, if the sEMG time-instant was classified as muscle activity (class 1);
- ${\widehat{y}}_{LSTM-MAD}$ = 0, if the sEMG time-instant was classified as background noise (class 0).

### Post-processing

**Fig. 3:**
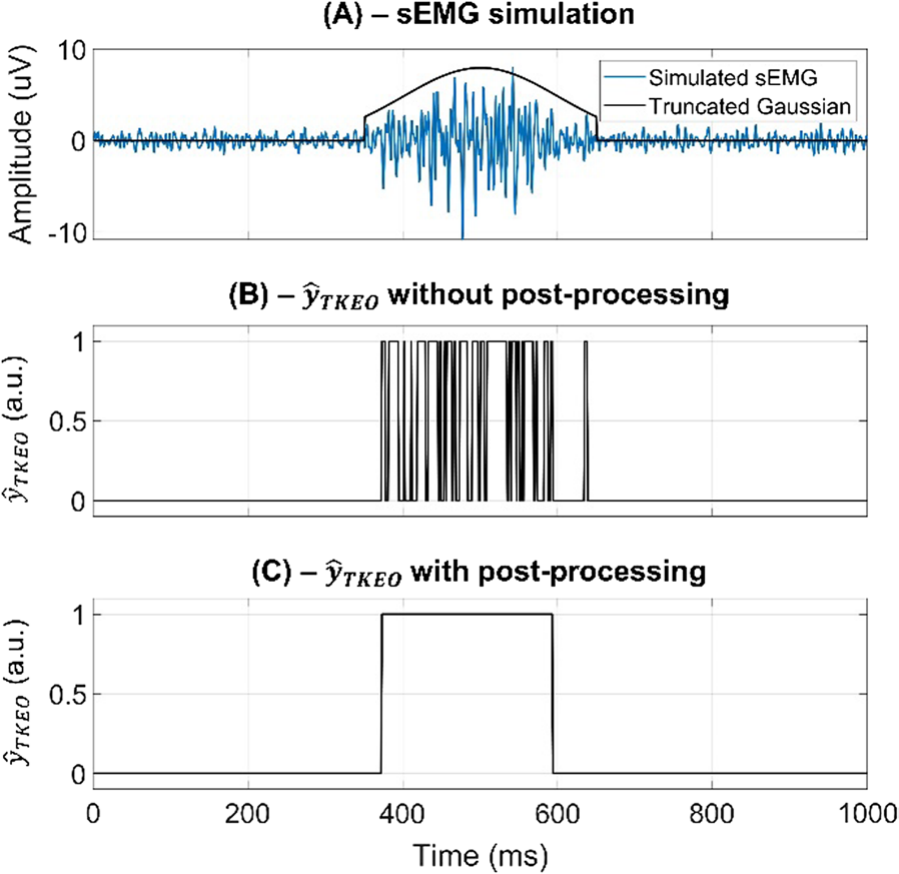
Example of post-processing applied to the standard approach output (${\widehat{y}}_{TKEO}$). **A** Sample realization of simulated sEMG signal (blue line) with the superimposition of the truncated Gaussian function used to modulate the muscle activity (black line). The SNR is set equal to 20 dB, the standard deviation of the truncated Gaussian (σ) is 100 ms, and the multiplicative constant (α) of the time support is 1.5. **B** The output of the standard approach (${\widehat{y}}_{TKEO}$) without any post-processing. **C** The output of the standard approach after rejecting all the activations shorter than 30 ms (post-processing step)

A post-processing step was applied to the output of each detector (i.e., standard, statistical, and deep learning approach) to reject the erroneous transitions due to the stochastic nature of the sEMG signal. Since it is generally accepted that a muscle activation shorter than 30 ms does not affect the kinetics and the kinematics of gait [48], all the muscle activations lasting less than 30 ms were discarded [27]. Figure 3 illustrates this concept. In particular, Fig. 3A shows a sample realization of a simulated sEMG signal modulated by a truncated Gaussian function (SNR = 16 dB, σ = 100 ms, and α = 1.5). Figure 3B represents the output of the standard approach (${\widehat{y}}_{TKEO}$) without any post-processing step, while Fig. 3C shows the effect of the post-processing on the detector’s output.

### Performance evaluation

The muscle activations detected by the three different approaches (${\widehat{y}}_{TKEO}$, ${\widehat{y}}_{Stat}$, and ${\widehat{y}}_{LSTM-MAD}$) were quantitatively compared against the ground truth in terms of precision, recall, F1-score, Jaccard similarity index, and onset/offset bias. More specifically, the indexes were defined as it follows:

6 $$precision= \frac{TP}{TP+FP}$$

7 $$recall= \frac{TP}{TP+FN}$$

8 $$F1-score= \frac{2 \times (recall \times precision)}{(recall+precision)}$$

9 $$Jaccard= \frac{\left|{\widehat{y}}_{i}\cap y\right|}{\left|{\widehat{y}}_{i}\cup y\right|}$$

10 $$Bias= \frac{1}{N}{\sum }_{i=1}^{N}\left({\widehat{t}}_{i}-t\right)$$

where $TP$ represents the True Positive (i.e., number of sEMG time-instants correctly classified by the detectors as muscle activity), $FN$ describes the False Negative (i.e., number of sEMG time-instants incorrectly classified by the detectors as background noise), and $FP$ represents the False Positive (i.e., number of sEMG time-instants incorrectly classified by the detectors as muscle activity). $y$ represents the binary mask computed by the $i$ th detector ($i$ = 1: standard approach with TKEO, $i$ = 2: statistical approach with the double-threshold statistical detector, $i$ = 3: novel approach with LSTM-MAD), and ${\widehat{y}}_{i}$ represents the ground truth of the simulated (or real) test set. Finally, $N$ is the number of estimates, ${\widehat{t}}_{i}$ is the estimate of the onset/offset time-instants obtained through the $i$ th detector, and $t$ represents the true onset/offset timings.

### Effect of SNR on muscle activity detection

The effect of sEMG signals’ SNR on the performance of the three tested muscle activity detectors was assessed considering the simulated test set by computing the performance parameters above-mentioned, separately for each of the nine SNR values (SNR = 3, 6, 10, 13, 16, 20, 23, 26, and 30 dB).

### Statistical analysis

The hypothesis of normality of the distribution of the computed performance parameters was tested through the Lilliefors test (“lillietest” MATLAB® function) setting the significance level (α) at 0.05. If the normality hypothesis was satisfied, one-way analysis of variance (ANOVA) for repeated measures (α = 0.05) was performed to assess significant differences in the performance of the three tested approaches and to test the effect of SNR on detectors’ performance, otherwise the Friedman’s test (α = 0.05) was implemented. Then, post-hoc analysis with Tukey’s adjustment for multiple comparisons was performed. The effect size of the statistically significant differences was calculated through the Hedges' *g* statistic [49] including the correction for small sample sizes. The statistical analysis was performed using the Statistical and Machine Learning Toolbox of MATLAB® release R2020b.

## Results

First, we present the results supporting the applicability of the LSTM-based approach for muscle activity detection, considering only simulated sEMG signals. Second, we further compare the performance of the three tested approaches (standard, statistical, and deep learning approach) on simulated sEMG signals, highlighting the effect of the SNR. Finally, we present the architecture and the performance of the LSTM-MAD model applied on the real sEMG signals.

### Simulated data

#### LSTM model definition

The best LSTM model was selected among all the tested networks as the one with the highest overall classification accuracy on the simulated test set, discarding those networks with a difference between the training and validation accuracy higher than 4% (to avoid overfitting of the training data).

Table 1 shows the properties of the LSTM model that achieved the highest overall classification accuracy (96.8% ± 4.3%) on the test set.

**Table 1:** Properties of the best LSTM model

LSTM layers	Properties
Sequence input layer	1 input feature
LSTM layer #1	275 hidden units Bi-directional sequence-to-sequence architecture
LSTM layer #2	138 hidden units Bi-directional sequence-to-sequence architecture
Fully connected layer	2units
Softmax layer	Softmax activation function (threshold = 0.5)
Classification output layer	2 classes (1 = muscle activity, 0 = background noise)

*LSTM* long short-term memory

### Performance evaluation

The performance of the three different muscle activity detectors was assessed and compared in terms of (i) precision, (ii) recall, (iii) F1-score, (iv) Jaccard similarity index, and (*v*) onset/offset bias:

i. *Precision:* An average precision of 0.92 ± 0.10, 0.98 ± 0.09, and 0.95 ± 0.08 was found on the simulated test set, considering the standard, the statistical, and the deep learning approach, respectively. Friedman’s test followed by post-hoc analysis revealed significant differences between each pair of detectors (*p* < 0.0001, *g* > 0.34);
ii. *Recall:* On average, a recall of 0.86 ± 0.20, 0.53 ± 0.39, and 0.96 ± 0.08 was found on the simulated test set, considering the standard, the statistical, and the deep learning approach, respectively. Friedman’s test followed by post-hoc analysis revealed significant differences between each pair of detectors (*p* < 0.0001, *g* > 0.66);
iii. *F1-score:* On average, an F1-score of 0.87 ± 0.14, 0.76 ± 0.25, and 0.95 ± 0.06 was found on the simulated test set, considering the standard, the statistical, and the deep learning approach, respectively. Friedman’s test followed by post-hoc analysis revealed significant differences between each pair of detectors (*p* < 0.0001, *g* > 0.54);
iv. *Jaccard similarity index:* An average Jaccard index of 0.80 ± 0.19, 0.52 ± 0.38, and 0.91 ± 0.10 was found on the simulated test set, considering the standard, the statistical, and the deep learning approach, respectively. Friedman’s test followed by *post-hoc* analysis revealed significant differences between each pair of detectors (*p* < 0.0001, g > 0.72);
v. *Onset/offset bias:* The onset/offset bias averaged over the simulated test set for each tested muscle activity detector are represented in Table 2. Considering the onset bias, Friedman’s test followed by *post-hoc* analysis revealed significant differences between each pair of detectors (*p* < 0.0001, g > 0.48). Even considering the offset bias, statistically significant differences were tested through Friedman’s test between each pair of detectors (*p* < 0.0001, g > 0.58);

**Table 2:** Onset/offset bias averaged over the simulated/real test set, for each muscle activity detector

	Onset bias (ms)			Offset bias (ms)		
	TKEO	Stat detector	LSTM-MAD	TKEO	Stat detector	LSTM-MAD
Simulated test set	24.3 ± 1.2	56.2 ± 2.2	4.0 ± 0.8	− 22.4 ± 1.2	− 65.1 ± 2.2	0.03 ± 0.7
Real test set	− 4.5 ± 16.1	35.6 ± 9.8	4.1 ± 2.2	19.2 ± 5.1	− 33.7 ± 12.1	− 5.8 ± 2.3

Values of parameters are reported as mean ± standard error over the simulated (and real) test set

*TKEO* Teager–Kaiser Energy Operator, *Stat detector* statistical double threshold detector, *LSTM-MAD* long short-term memory muscle activity detector

**Fig. 4:**
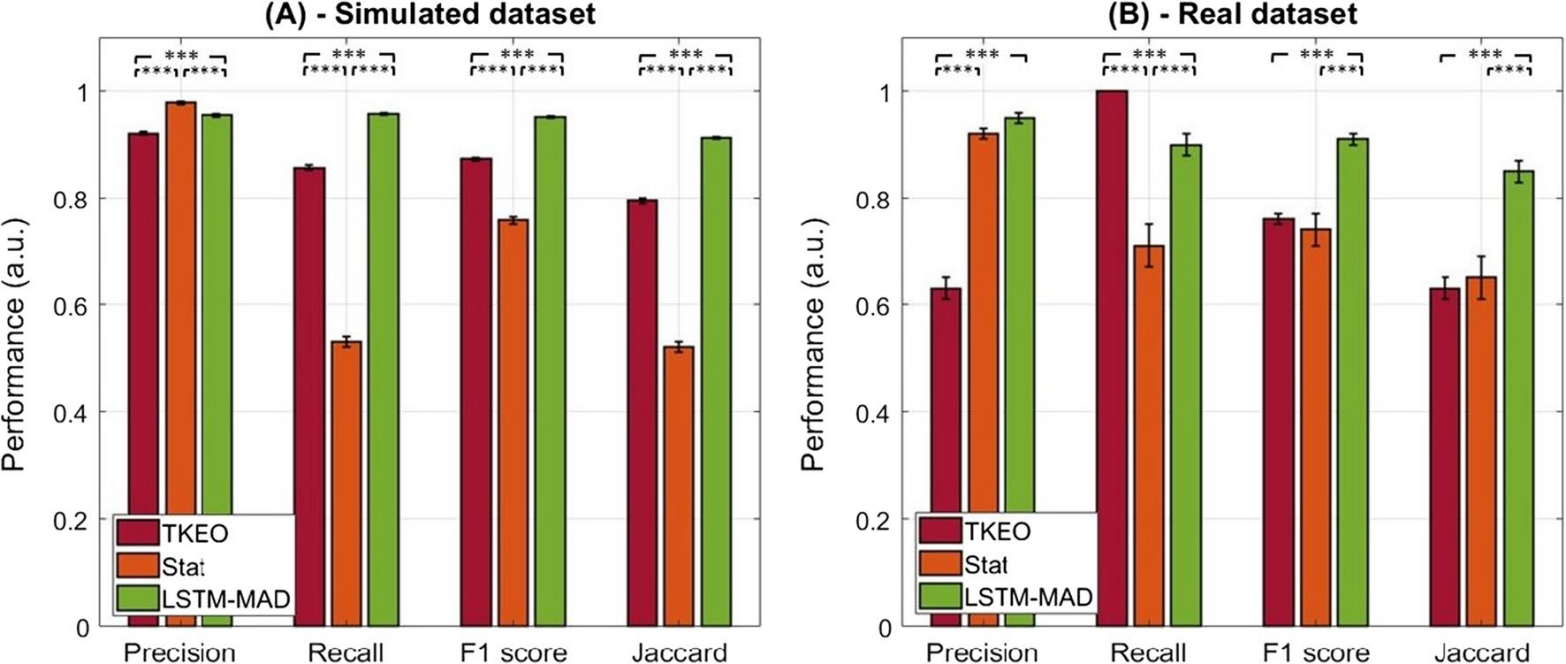
Comparison of the performance of the three muscle activity detectors (standard TKEO by Li et al*.* [29], statistical double-threshold detector by Bonato et al*.* [27], and LSTM-MAD) estimated considering (**A**) the simulated and (**B**) the real dataset. Average values and standard errors are represented. Statistically significant differences are indicated by asterisks (***p < 0.0001)

Figure 4A compares the performance of the three tested detectors (standard, statistical, and deep learning approach) in terms of precision, recall, F1-score, and Jaccard similarity index. The average values and standard errors of these parameters were estimated on the simulated test set. Asterisks highlight statistical differences (*p* < 0.05) between each pair of detectors.

### Effect of SNR on muscle activity detection

The effect of the SNR on the detector performance was assessed by extracting from the simulated test set the above-mentioned performance parameters (precision, recall, F1-score, Jaccard similarity index, and onset/offset bias), separately for each simulated SNR-value.

**Fig. 5:**
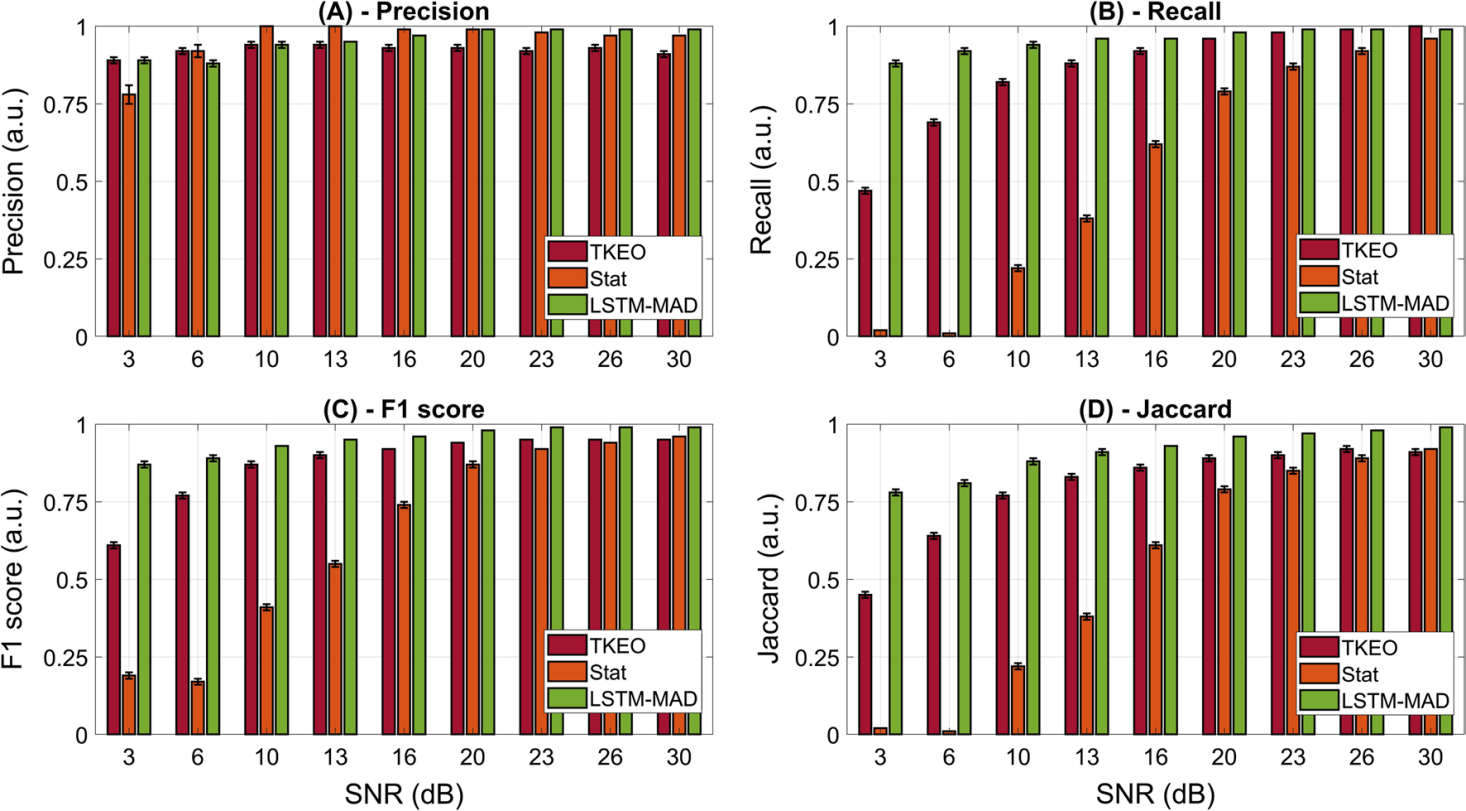
Values of **A** precision, **B** recall, **C** F1 score, and **D** Jaccard similarity index, averaged on the simulated test set, for each value of SNR and for each muscle activity detector. Each colored bar represents the average performance of a specific detector (TKEO by Li et al*.* [29] in red, Stat detector by Bonato et al*.* [27] in orange, and LSTM-MAD in green). Error bars represent the standard errors

Figure 5 represents, for each muscle activity detector, the average values (and standard errors) of precision (Fig. 5A), recall (Fig. 5B), F1-score (Fig. 5C), and Jaccard similarity index (Fig. 5D), for each simulated SNR-value. For all these parameters, LSTM-MAD revealed a higher performance consistency across the different SNR values, suggesting a lower effect of SNR on muscle activity detection compared to the other two approaches. As expected, the approach more affected by the SNR was the Stat, with an evident decrease in the performance parameters for simulated sEMG signals with SNR values lower than 20 dB.

Table 3, instead, shows the onset/offset bias values averaged over the simulated test set for each simulated SNR value. Even considering the onset and offset bias, LSTM-MAD revealed higher performance across the different SNR values with (absolute) onset/offset bias values significantly lower compared to the other two approaches. Among the three testes approaches, the worst performance was achieved by the Stat approach which revealed a significant increase in the (absolute) onset/offset bias values for simulated sEMG signals with SNR values lower than 20 dB.

**Table 3:** Onset/offset bias averaged over the simulated test set for each value of SNR and for each muscle activity detector

SNR	Onset bias (ms)			Offset bias (ms)		
	TKEO	Stat detector	LSTM-MAD	TKEO	Stat detector	LSTM-MAD
30 dB	− 4.5 ± 0.8	1.1 ± 1.4	0.8 ± 0.2	7.8 ± 1.0	− 10.7 ± 1.6	0.6 ± 0.3
26 dB	− 2.3 ± 0.73	10.6 ± 2.3	− 0.2 ± 0.4	3.2 ± 1.0	− 20.6 ± 2.3	0.1 ± 0.4
23 dB	0.2 ± 1.1	23.2 ± 2.9	− 0.72 ± 0.7	0.7 ± 1.1	− 29.8 ± 2.6	− 0.6 ± 0.6
20 dB	2.8 ± 1.3	36.4 ± 3.4	1.1 ± 0.9	− 4.8 ± 1.3	− 46.4 ± 3.5	− 2.2 ± 0.9
16 dB	15.4 ± 2.1	67.1 ± 4.7	2.9 ± 1.5	− 13.9 ± 1.9	− 76.5 ± 4.5	2.5 ± 1.7
13 dB	21.0 ± 2.6	105.0 ± 5.6	1.5 ± 2.0	− 20.5 ± 2.7	− 109.9 ± 5.9	− 0.4 ± 1.9
10 dB	33.0 ± 3.2	129.0 ± 7.5	3.4 ± 2.6	− 35.1 ± 3.3	− 139.8 ± 7.88	− 2.8 ± 2.2
6 dB	59.1 ± 4.2	200.3 ± 21.8	6.6 ± 3.3	− 55.6 ± 4.4	− 202.2 ± 21.8	4.5 ± 3.3
3 dB	86.3 ± 5.6	171.3 ± 19.3	20.8 ± 4.4	− 83.9 ± 5.3	− 196.6 ± 15.3	− 1.9 ± 3.5

Values of parameters are reported as mean ± standard error over the simulated test set

*TKEO* Teager–Kaiser Energy Operator, *Stat Detector* statistical double threshold detector, *LSTM-MAD* long short-term memory muscle activity detector, *SNR* signal-to-noise ratio

### Real data

The same procedure described for the simulated dataset was followed to define and select the best LSTM model considering real sEMG data. In the following, the results obtained from the real dataset for the three different approaches are detailed.

### LSTM model definition

The same 720 different LSTM models considered for the simulated dataset were tested considering the real data. The best LSTM model that achieved the highest overall classification accuracy (90.1% ± 14.28%) on the test set revealed the same architecture and properties as the one selected considering the simulated data (see Table 1).

**Fig. 6:**
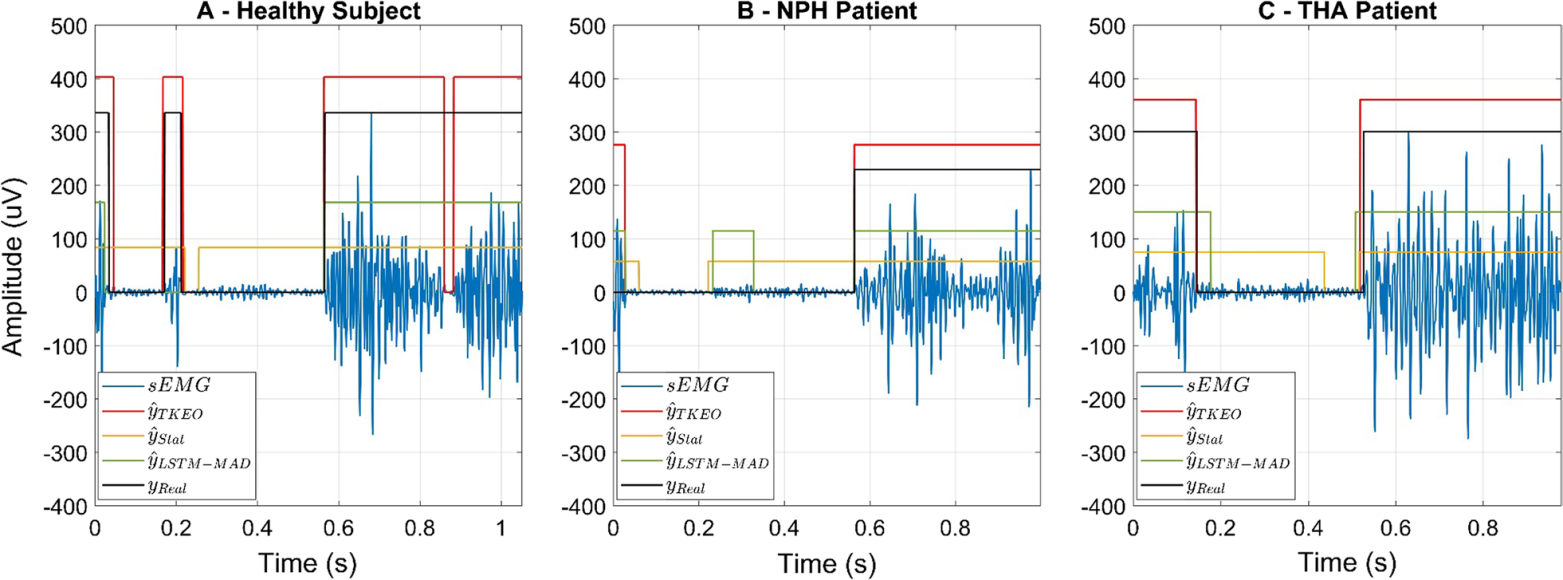
Example of real sEMG signals (blue lines) acquired from the TA muscle of **A** a healthy subject, **B** a patient affected by idiopathic Normal Pressure Hydrocephalus (NPH), and **C** a patient who underwent a unilateral Total Hip Arthroplasty (THA). The output of the standard (red lines), statistical (orange lines), and LSTM-MAD approach (green lines) are represented along with the indication of the ground truth (black lines) manually segmented by expert operators. All the muscle activities shorter than 30 ms have been rejected by means of the post-processing step for all the approaches

Figure 6 shows an example of a real sEMG signal acquired from the TA muscle of a healthy subject of the sample population with the superimposition of the ground truth (${y}_{Real}$) and the outputs of the three detectors (${\widehat{y}}_{TKEO}$, ${\widehat{y}}_{Stat}$, and ${\widehat{y}}_{LSTM-MAD}$) after the post-processing step.

### Performance evaluation

The performance of the three different muscle activity detectors on the real testset was assessed considering the same five parameters described for the simulated sEMG signals:

i. *Precision:* An average precision of 0.63 ± 0.15, 0.92 ± 0.11, and 0.95 ± 0.11 was found on the real test set, considering the standard, the statistical, and the deep learning approach, respectively. Friedman’s test followed by post-hoc analysis revealed significant differences between the standard and the statistical approach (*p* < 0.0001, *g* = 2.20), and between the standard and the deep learning approach (*p* < 0.0001, *g* = 2.43), while no difference was found between the statistical and the deep learning approach (*p* = 0.92);
ii. *Recall:* On average, a recall of 1.00 ± 0.00, 0.71 ± 0.31, and 0.90 ± 0.15 was found on the real test set, considering the standard, the statistical, and the deep learning approach, respectively. Friedman’s test followed by post-hoc analysis revealed significant differences between each pair of detectors (*p* < 0.04, *g* > 0.78);
iii. *F1-score:* On average, an F1-score of 0.76 ± 0.11, 0.74 ± 0.26, and 0.91 ± 0.11 was found on the real test set, considering the standard, the statistical, and the deep learning approach, respectively. Friedman’s test followed by post-hoc analysis revealed significant differences between the standard and the deep learning approach (*p* < 0.0001, *g* = 1.36), and between the statistical and the deep learning approach (*p* < 0.0001, *g* = 0.85), while no difference was found between the standard and the statistical approach;
iv. *Jaccard similarity index:* An average Jaccard index of 0.63 ± 0.15, 0.65 ± 0.27, and 0.85 ± 0.16 was computed from the real test set, considering the standard, the statistical, and the deep learning approach, respectively. Friedman’s test followed by post-hoc analysis revealed significant differences between the standard and the deep learning approach (*p* < 0.0001, *g* = 1.42), and between the statistical and the deep learning approach (*p* < 0.0001, *g* = 0.90), while no difference was found between the standard and the statistical approach;
v. *Onset/offset bias:* The onset/offset bias averaged over the real test set for each tested muscle activity detector are represented in Table 2. Considering the onset bias, Friedman’s test followed by post-hoc analysis revealed significant differences between the standard and the statistical approach (*p* = 0.04, *g* = 0.48) and between the standard and the deep learning approach (*p* = 0.02, *g* = 0.57), while no difference was found between the statistical and the deep learning approach (*p* = 0.74). Considering the offset bias, statistically significant differences were tested between each pair of detectors (*p* < 0.0001, g > 0.21).

Considering the real test set, Fig. 4B compares the performance of the three detectors (standard, statistical, and deep learning approach) in terms of precision, recall, F1-score, and Jaccard similarity index. The average values and standard errors of these parameters are reported, as well as asterisks to highlight statistical differences (*p* < 0.05) between each pair of detectors.

Additional details on the performance assessment conducted considering real data can be found in the supplementary material (see Additional file 1). In particular, data (accuracy, precision, recall, F1 score, and SNR) are reported, separately, for each subject of the sample population (labeled as “healthy”, “THA”, or “NPH”) and each muscle analyzed (RF, LH, LGS, and TA). Bar diagrams comparing the three detectors are also provided considering separately sample populations (Additional file 1: Figure S1) and muscles (Additional file 1: Figure S2).

## Discussion

Results presented in this work demonstrated that muscle activity detection during gait can be successfully performed using the novel approach based on Long Short-Term Memory (LSTM) Recurrent Neural Networks (RNNs). The newly introduced LSTM-MAD was proven to outperform the tested state-of-the-art approaches and effectively separate activation intervals from background noise, with an overall classification accuracy of 97% (simulated data) and 90% (real data). More specifically, LSTM-MAD clearly exhibits better performance than both the alternative approaches tested (standard approach using the Teager-Keiser Energy Operator (TKEO) [29], and double-threshold statistical detector (Stat) [27]).

In the last decades, the extraction of the onset/offset timing of the muscular activity from sEMG signals has found a great interest in different research areas, including neurorobotics and myoelectric control of prostheses [3], motor rehabilitation and sport science [2], and human–machine interaction [4]. Accordingly, several approaches have been proposed in the literature to extract the onset and offset time-instants of muscle activations during human movements [12, 15–26]. The majority of the published detectors are threshold-based approaches, such as the single-threshold detector based on the Teager–Kaiser Energy Operator [29, 30] and the double-threshold statistical detector proposed by Bonato et al*.* [27]. However, these methods suffer from two main limitations: (i) the extraction of the onset/offset time instants rely on the extraction of time- and frequency-domain features which may not be sufficient to properly assess dynamic muscle activity and (ii) their performance are strongly affected by the amount of noise superimposed to the sEMG signal. Even if different studies proposing models able to efficiently work even at very low SNR of sEMG signals have been published in the last years [13, 31, 50], these methods still suffer from the first of the above-mentioned limitations (i.e., the necessity of a feature extraction step before muscle activation interval detection).

In the last years, Long Short-Term (LSTM) and Gated Recurrent Units (GRU) [17, 51–54] Recurrent Neural Networks (RNNs) have been proposed, in addition to the traditional approaches, to classify and recognize human movements starting from sEMG signals by exploiting the ability of these networks to recognize patterns and time-dependencies in long sequential data. Even if GRU recurrent neural networks require less computational cost compared to the LSTM recurrent neural networks, several studies demonstrated that LSTM-RNNs outperform the GRU-RNNs [51–54]. The detection of the start and end time-instants of muscle activations during human movements can be considered itself a classification problem, where each sample of the sEMG signals should be classified as active (presence of muscle activation) or non-active (absence of muscle activation). The application of LSTM (or GRU) neural networks to the muscle activation detection problem could result in better performance compared to the previously published approaches by taking advantage of their ability to learn time dependencies in long sequential data (e.g., sEMG signals).

In this work, a novel approach, based on a LSTM-RNN model, for the detection of muscle activation intervals was presented and validated on both simulated and real sEMG signals to overcome the main limitations of the previously published approaches. The newly introduced muscle activity detector (LSTM-MAD) was proven to effectively separate activation intervals from background noise considering both simulated and real sEMG signals. The performance of the three tested approaches on the real sEMG signals was tested against a ground truth defined by three expert operators that visually segmented the real data through a custom MATLAB® GUI. Although it is acknowledged that the visual definition of the onset/offset timing is not optimal and highly subjective [12], to the best of the authors’ knowledge, it currently provides the only gold-standard procedure that allows for comparing the performance of different approaches considering real sEMG data. Moreover, several studies demonstrated the inaccuracy of the visual onset/offset determination to be lower than ± 5–10 ms [55–58]. Our study confirmed this finding on the operators’ performance, highlighting the great potential of the proposed LSTM-MAD method. Indeed it allows for obtaing a performance comparable to that of expert operators, without the need for cumbersome and time-consuming sessions of manual segmentation.

Considering simulated sEMG signals, all the performance parameters introduced (precision, recall, F1-score, Jaccard similarity index, and onset/offset bias) showed remarkably better values for LSTM-MAD when compared to the Stat approach. Furthermore, greater values of recall, F1-score, and Jaccard similarity index were found for LSTM-MAD when compared to the TKEO detector, and only a slightly worst precision. However, while LSTM-MAD shows an excellent balance between precision and recall, the same cannot be said for the TKEO detector, which displays a very high precision (i.e., low number of false-positive classifications), but a very low recall (i.e., high number of false-negative classifications). In other words, the TKEO detector revealed a reduced probability of detection and an increased number of false-negative classifications compared to the deep learning approach. Indeed, this “optimal balance” between precision and recall is incorporated in the definition of the other two parameters (F1-score and Jaccard similarity index), which are broadly used in literature specifically to take into account this important aspect.

Considering real signals, all the performance parameters showed remarkably better values for LSTM-MAD compared to the TKEO detector. Furthermore, greater values of precision, F1-score, and Jaccard similarity index were found for LSTM-MAD when compared to the TKEO detector. Only the recall was higher in the TKEO detector compared to our approach. Again, it should be noted that LSTM-MAD is characterized by an excellent balance between precision and recall, while this is not true for the TKEO and Stat detectors. Indeed, the TKEO detector shows a very good recall to the detriment of very poor precision. The Stat detector, instead, showed a different behavior, revealing a very high Precision (similar to the one obtained considering the LSTM-MAD approach) to the detriment of a very poor recall. In other words, the Stat detector demonstrated a reduced probability of detection and an increased number of false-negative classifications compared to the LSTM-MAD approach.

Overall, LSTM-MAD revealed a smaller variability in the detector’s performance, especially compared to the TKEO approach. Although a thorough analysis of this aspect is beyond the scope of this work, this reduced variability can be qualitatively appreciated in Fig. 4, when comparing the small error bars obtained for LSTM-MAD compared to those obtained for TKEO.

The novel approach introduced in this work revealed increased robustness of the detector’s performance compared to the effect of the SNR, suggesting the applicability of the LSTM-MAD to a wider range of noise conditions compared to the other two tested approaches. Indeed, while it is evident that decreasing SNR inevitably diminishes the detection performance of each approach, LSTM-MAD is the least affected one (see Fig. 5). In particular, focusing on the parameters recall, F1-score, and Jaccard similarity index, we found a remarkable worsening of the performance of the Stat detector with decreasing SNR. The situation is even more dramatic considering the TKEO detector. On the contrary, the LSTM-MAD detector shows a limited worsening of the performance with decreasing SNR. Indeed, even at very low SNR values (e.g., 3 or 6 dB), the performance of LSTM-MAD never degrades too much (recall is always greater than 0.88, F1-score is always greater than 0.87, and Jaccard similarity index is always greater than 0.78). For what concerns the parameter precision, none of the three detectors showed a drastic decrease of performance with decreasing SNR value. The analysis about how a poor SNR can eventually degrade detectors’ performance was carried out on simulated sEMG signals only. This was chosen to study the above-described phenomenon in a more controlled condition, i.e., to have a precise knowledge (a priori) about the SNR itself (since sEMG signals were simulated at each specific SNR level). Indeed, considering real signals one would have needed to apply some additional algorithms to estimate the SNR value.

Another valuable attribute and distinctive quality of the LSTM-MAD approach is that it does not require any additional input parameters, such as the background-noise power, SNR, and signal energy or amplitude. Our algorithm directly works on “raw” sEMG signals, the only pre-processing step being the usual bandpass filtering (between 10 and 450 Hz) [40], applied in all three approaches, in the same manner. On the contrary, the double-threshold statistical detector requires, as a necessary input parameter, the knowledge of the background-noise power and the SNR. The estimation of background-noise power and SNR is usually obtained by analyzing 30 s-windows of sEMG signal through dedicated algorithms, such as the one proposed by Agostini et al*.* [28]. However, since LSTM-MAD does not require any additional input parameter (e.g., background-noise power or SNR), it is intrinsically more adaptable to eventual SNR variations arising during signal acquisition.

A further advantage of the proposed approach is that it allows for obtaining a more accurate and precise detection of the muscle activity timing in terms of onset and offset (absolute) bias compared to the other two tested threshold-based approaches. In particular, LSTM-MAD revealed an average estimated bias lower than 6 ms in the onset/offset of muscle activation intervals, on both simulated and real sEMG signals, suggesting the method's applicability to both basic research and clinical practice [12, 27].

The detection of the muscle activation intervals during human movements can also be strongly affected by the presence of spurious background spikes, in particular considering pathological conditions, such as a paretic forearm and hand [32–34]. However, due to the lack of availability in our database of sEMG signals acquired from stroke survivors or spinal cord injury patients (whose sEMG signals are often characterized by this kind of background noise), no specific analyses were performed in this work on this aspect. Further studies could be focused on the assessment of the effect of the spurious background noise on the performance of the newly introduced muscle activity detector by analyzing its ability to distinguish between voluntary sEMG spurious spikes.

There are two main limitations in this study. The first one is the small sample size (20 subjects) of the real dataset used to assess the applicability of the LSTM-based approach to gait analysis, while the second is that only sEMG signals acquired during walking were considered. However, a further step could be the application of this novel approach on sEMG signals acquired during movements other than walking (e.g., non-cyclical movements) and from subjects with different musculoskeletal or neurological disorders (e.g., Parkinson’s disease or post-stroke) to assess its applicability also in different pathological conditions.

## Conclusions

We proposed and validated, both on simulated and real sEMG signals, a Long Short-Term Memory approach for muscle activity detection. The presented approach clearly outperforms alternative detectors (the standard approach based on Teager–Kaiser Energy Operator (TKEO) and the double-threshold statistical detector (Stat)), revealing an onset/offset (absolute) bias smaller than 6 ms, and a good performance even for signals with low to medium SNR. The results of this work demonstrated that the proposed detector can be considered a valuable tool, suitable to all the applications requiring an accurate and effective recognition/distinction of muscle activity from background noise in sEMG signals, such as gait analysis, motor rehabilitation, and myoelectric control of prostheses.

## Supplementary Information

**Additional file 1.** Detectors’ performance on real data. Performance of the three tested approaches in terms of overall classification accuracy, precision, recall, F1 score, and Jaccard similarity index, divided by sample population and muscle acquired. **Figure S1.** Sample populations. **Figure S2.** Muscles.

## Data Availability

The real data presented in this study to train and test the LSTM-MAD can be found online at Zenodo (https://doi.org/10.5281/zenodo.4391062) in MAT and CSV format.

## Abbreviations

ANOVA: Analysis of variance
LGS: Lateral gastrocnemius
LH: Lateral hamstring
LSTM: Long short-term memory neural network
LSTM-MAD: Long short-term memory-based muscle activity detection
NPH: Normal pressure hydrocephalus
RF: Rectus femoris
RNN: Recurrent neural network
sEMG: Surface electromyography
SNR: Signal-to-noise ratio
Stat: Double-threshold statistical detector
TA: Tibialis anterior
THA: Total hip arthroplasty
TKEO: Teager–Kaiser Energy Operator
